# Survival benefit of enteral nutrition and airway interventions in multiple system atrophy

**DOI:** 10.1016/j.prdoa.2026.100499

**Published:** 2026-09-09

**Authors:** Katsuya Nishida, Kento Sakashita, Naonobu Futamura

**Affiliations:** a Department of Neurology, National Hospital Organization Hyogo Chuo National Hospital, 1314 Ohara, Sanda, Hyogo 669-1592, Japan

**Keywords:** Multiple system atrophy, Dysphagia, Enteral nutrition, Laryngotracheal separation, Advance care planning, Survival analysis

## Abstract

**Introduction:**

Dysphagia in multiple system atrophy (MSA) leads to pneumonia and malnutrition, impacting survival. This study evaluated the association between enteral nutrition (EN) and survival and described outcomes of laryngotracheal separation (LTS).

**Methods:**

We retrospectively analyzed 158 patients with probable/definite MSA (2000–2025). Survival from onset was evaluated using Simon–Makuch curves, Mantel–Byar tests with time-dependent EN, and Kaplan–Meier analysis with propensity score matching (PSM) and restricted mean survival time (RMST). Competing risk analyses (Fine–Gray models) assessed the cumulative incidence of death and respiratory interventions (tracheostomy or tracheostomy invasive ventilation [TIV]). Clinical outcomes of LTS (*n* = 11) were reviewed.

**Results:**

Eighty-seven patients received EN; median survival after EN initiation was 4.3 years. Simon–Makuch analysis showed lower survival in the EN group, reflecting late-stage introduction. After PSM, EN was associated with a modest survival advantage at 7 years (RMST difference 0.68 years, *P* = 0.005). Significant reduction in sudden death occurred in the EN group (15% vs. 30%, *P* = 0.001). Competing-risk models showed lower death and higher TIV incidence in the EN group, reflecting proactive respiratory management. In the LTS series, mean survival was 14.5 years with no pneumonia-related deaths and preserved limited oral intake, supporting quality-of-life benefits.

**Conclusion:**

EN was independently associated with a statistically significant but clinically modest survival advantage, largely by facilitating a comprehensive care pathway including respiratory management. In a small LTS series, the prevention of pneumonia-related death and potential quality-of-life benefits support early structured advance care planning in advanced MSA.

## Highlights

• Enteral nutrition was associated with a modest survival advantage in MSA.
• Competing risk analysis showed higher TIV incidence in the enteral nutrition group.
• Enteral nutrition facilitates a comprehensive care pathway in advanced MSA.
• Laryngotracheal separation prevented pneumonia death while maintaining oral intake.
• Structured advance care planning is crucial for managing dysphagia in MSA.

## 1 Introduction

Multiple system atrophy (MSA) is characterized by rapid progression, with dysphagia being a major complication affecting quality of life (QoL) and survival [1], [2]. Early dysphagia is associated with a more aggressive phenotype and shorter survival [3]. Regular assessment and timely intervention for swallowing dysfunction are therefore important in multidisciplinary care.

When oral intake becomes unsafe or insufficient, enteral nutrition (EN) via nasogastric tube or gastrostomy may be initiated to maintain nutrition and reduce aspiration risk [4], [5]. However, the optimal timing of EN and its association with survival remain uncertain, with concerns that it may not always translate into improved QoL in advanced stages. Although progressive dysphagia or airway obstruction may necessitate tracheostomy or tracheostomy invasive ventilation (T/TIV), their long-term survival benefit remains debated [5], [6].

When EN alone is insufficient to prevent aspiration, aspiration-prevention surgery, such as laryngotracheal separation (LTS), may be considered. By separating the airway from the digestive tract, LTS can reduce the risk of aspiration while preserving the laryngeal framework. Previous reports have suggested reduced aspiration pneumonia and the possibility of maintaining limited oral intake after surgery [7], [8]. Nevertheless, evidence regarding long-term survival and QoL after LTS in MSA remains sparse.

This study evaluated the association between EN and survival in patients with MSA receiving long-term inpatient care. We also examined invasive interventions, including LTS and T/TIV, in the context of advance care planning (ACP).

## 2 Methods

### 2.1 Participants and study design

This retrospective study included 158 patients with probable or definite MSA hospitalized at our institution between January 2000 and January 2025. Diagnosis was established according to the second consensus criteria [9], supported by clinical assessment of autonomic dysfunction, including orthostatic blood pressure changes and urinary dysfunction, and neuroimaging findings. Genetic testing for spinocerebellar ataxias was selectively performed when clinically indicated. EN was initiated by a multidisciplinary team based on videofluoroscopic swallowing studies (VFSS) or bedside evaluations when oral intake became unsafe or nutritionally inadequate.

### 2.2 Data collection

Clinical variables included age at onset, sex, clinical subtype (MSA-C/P), and disease milestones (indwelling bladder catheterization, EN, tracheostomy, and TIV). The primary outcome was overall survival from disease onset. Secondary outcomes included the time to EN, T/TIV initiation, and a composite endpoint of death or T/TIV. Sudden death was defined as unexpected, non-traumatic death within 1 h of acute symptom onset if witnessed, or within 24 h of being last seen clinically stable if unwitnessed.

### 2.3 Statistical analysis

To address immortal time bias, EN and T/TIV were treated as binary time-varying exposures. Survival was analyzed using Simon–Makuch plots and Mantel–Byar tests, truncated at τ = 7 years post-onset to ensure clinical comparability and avoid extrapolation [3], [6]. Propensity scores for EN initiation were calculated using age, sex, clinical subtype, and time to bladder catheterization as a proxy for disease progression. We performed 1:1 nearest-neighbor propensity score matching (PSM), with balance evaluated using standardized mean differences (SMD < 0.1).

Matched-cohort survival was assessed with Kaplan–Meier curves (log-rank test) and restricted mean survival time (RMST) at τ = 7 years. Further analyses included inverse probability of treatment weighting (IPTW) Cox models. To evaluate competing risks between death and T/TIV, we estimated cumulative incidence functions (CIFs) and fitted Fine–Gray subdistribution hazard models. A multivariable time-dependent Cox regression model was fitted, adjusting for age at onset, sex, clinical subtype (MSA-C vs. MSA-P), and time to bladder catheterization. Sensitivity analyses used similarly adjusted Cox models stratified by onset era (≤2013 vs. ≥2014), including an EN-by-era interaction test. EZR version 1.68 (Jichi Medical University, Saitama, Japan), a graphical user interface for R version 4.3.1, was used [10]. Two-sided *P* values <0.05 were considered significant.

### 2.4 Ethical considerations

This study was approved by our institutional review board (approval no. 20–05). Written informed consent was obtained whenever feasible; otherwise, an opt-out procedure was applied.

## 3 Results

### 3.1 Patient characteristics

Among the 158 patients (Table 1), 87 (55%) initiated EN. Compared with the non-EN group, the EN group was younger at onset (59.5 vs. 64.2 years, *P* = 0.002) and had a higher frequency of MSA-P (40% vs. 24%, *P* = 0.041). Urinary catheterization (89% vs. 47%) and tracheostomy (61% vs. 7%) were more frequent in the EN group (both *P* < 0.001). All TIV cases were initiated after EN. Overall, 125 patients (79.1%) died during follow-up. In the overall cohort (*n* = 158), causes of death included sudden death in 38 (24.1%), respiratory failure or arrest in 35 (22.2%), pneumonia in 18 (11.4%), other infections or urosepsis in 12 (7.6%), cardiovascular disease in 6 (3.8%), malignancy in 4 (2.5%), and other or unspecified causes in 12 (7.6%). Sudden death was more frequent in the non-EN group (32% vs. 17%, *P* < 0.001). The median survival from EN initiation was 4.3 years (95% confidence interval [CI] 2.8–5.2).

**Table 1:** Characteristics, interventions, and clinical outcomes before and after propensity score matching.

	**Total Cohort** **(*n* = 158)**		**Propensity-Matched** **Cohort (*n* = 106)**		
	Non-EN (*n* = 71)	EN (*n* = 87)	Non-EN (*n* = 53)	EN (*n* = 53)	
**Baseline characteristics**					**SMD**^**⁎**^
Age at onset, y	64.2	59.5	62.6	61.5	0.116
Sex, male, *n* (%)	36 (51)	46 (53)	33 (62)	32 (60)	0.039
MSA subtype					0.084
MSA-P, *n* (%)	17 (24)	35 (40)	16 (30)	14 (26)	
MSA-C, *n* (%)	54 (76)	52 (60)	37 (70)	39 (74)	
Time to urinary catheterization, y	5.5	7.1	5.7	5.7	0.001

**Clinical interventions**					
Time to EN initiation, y	–	7.0	–	6.5	
Tracheostomy, *n* (%)	5 (7)	53 (61)	2 (4)	37 (70)	
TIV, *n* (%)	0 (0)	22 (25)	0 (0)	17 (32)	
LTS⁎⁎, *n* (%)	2 (3)	9 (10)	1 (2)	7 (13)	

**Clinical outcomes**					***P* value**^⁎^
Overall survival, y	6.4	11.9	6.7	10.1	0.018
Sudden death, *n* (%)	23 (32)	15 (17)	16 (30)	8 (15)	0.001
Pneumonia death, *n* (%)	6 (8)	12 (14)	6 (11)	4 (8)	0.610

**Statistical analyses**	**Estimate**		**95% CI**		***P* value**
*Main analysis*					
RMST difference, y	0.68		0.23–1.12		0.005

*Sensitivity analyses*					
Time-dependent Cox (HR)	0.74		0.57–0.96		0.024
IPTW-weighted Cox (HR)	0.73		0.58–0.91		0.006
Competing risk (SHR)	0.67		0.51–0.88		0.004

**Footnotes:**

**Abbreviations:** CI, confidence interval; EN, enteral nutrition; HR, hazard ratio; IPTW, inverse probability of treatment weighting; LTS, laryngotracheal separation; PSM, propensity score matching; RMST, restricted mean survival time; SHR, subdistribution hazard ratio; SMD, standardized mean difference; TIV, tracheostomy invasive ventilation.

**Statistical notes:***P* values were calculated using the *t*-test or Mann–Whitney *U* test for continuous variables and the chi-square test or Fisher's exact test for categorical variables, as appropriate. Continuous variables are presented as mean for baseline characteristics and median for survival data. SMDs are shown for baseline characteristics in the propensity-matched cohort. ^**⁎**^SMD and *P* values in the baseline characteristics and clinical outcomes sections were calculated within the propensity-matched cohort.

⁎⁎ **LTS Subgroup** (*n* = 11): Mean overall survival was 14.5 years; no deaths were attributed to pneumonia. Deaths (*n* = 4) were due to sudden death (*n* = 1), heart failure (*n* = 1), malignancy (*n* = 1), and urinary tract infection (*n* = 1). Detailed clinical data are provided in Supplementary Table material.

### 3.2 Survival and competing-risk analyses

**Fig. 1:**
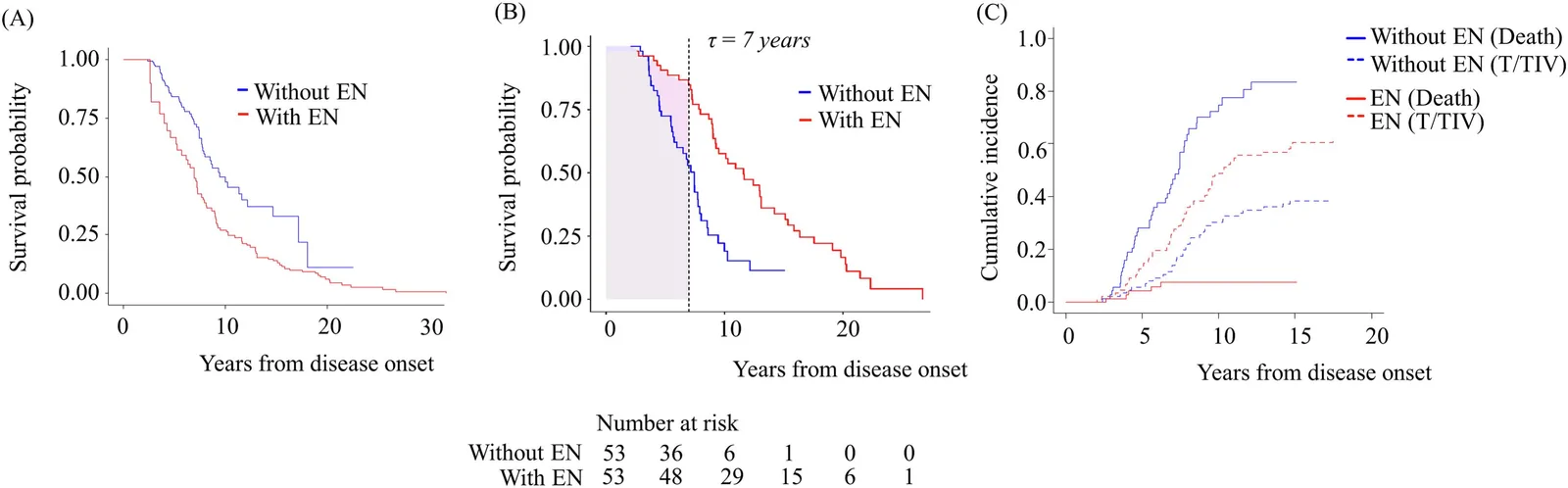
Survival and competing-risk analyses in patients with multiple system atrophy (MSA) according to enteral nutrition (EN). **(A)** Simon–Makuch plot with EN modeled as a time-varying exposure; patients contribute person-time to the non-EN group until EN initiation. The lower EN curve likely reflects late EN initiation. Group differences were assessed with the Mantel–Byar test (*P* < 0.001). **(B)** Kaplan–Meier curves after propensity score matching. Survival differed between groups (log-rank *P* = 0.041), with a modest restricted mean survival time difference of 0.68 years at τ = 7 years (*P* = 0.005). **(C)** Fine–Gray competing-risk analysis of cumulative incidence of death and tracheostomy/tracheostomy invasive ventilation (T/TIV) according to EN. EN was associated with a lower cumulative incidence of death (subdistribution hazard ratio [SHR] 0.67, *P* = 0.004) and a higher cumulative incidence of T/TIV (SHR 1.46, *P* = 0.009). Note the reversal of cumulative incidence functions between groups, suggesting a shift in the clinical care pathway.

In analyses truncated at τ = 7 years, Simon–Makuch curves showed lower survival in the EN group (Mantel–Byar *P* < 0.001), reflecting late EN initiation (Fig. 1A). However, after propensity-score matching (Table 1), the EN group demonstrated longer survival (log-rank *P* = 0.041; Fig. 1B). In the matched cohort, sudden death was less frequent in the EN group (15% vs. 30%, *P* = 0.001), while pneumonia-related death did not differ (8% vs. 11%, *P* = 0.610). The RMST difference was 0.68 years (95% CI, 0.23–1.12; *P* = 0.005). An IPTW-weighted Cox model showed lower mortality associated with EN (hazard ratio [HR] 0.73; 95% CI 0.58–0.91; *P* = 0.006).

Fine–Gray models revealed that the EN group had a lower cumulative incidence of death (subdistribution HR [SHR] 0.67; *P* = 0.004) and a higher cumulative incidence of T/TIV (SHR 1.46; *P* = 0.009). The CIF for death and T/TIV reversed between groups (Fig. 1C); respiratory interventions tended to precede death in the EN group, whereas death was more frequent without prior T/TIV in the non-EN group. Multivariable time-dependent Cox regression showed that both EN (adjusted HR [aHR] 0.74, *P* = 0.024) and T/TIV (aHR 0.62, *P* = 0.003) were associated with lower mortality. In sensitivity analyses by onset era, EN was associated with lower mortality in the early era (≤2013; aHR 0.36, 95% CI 0.22–0.62), while the association was directionally similar in the late era (≥2014; aHR 0.64, 95% CI 0.25–1.65), with no significant EN-by-era interaction (*P* = 0.113).

### 3.3 LTS subgroup

LTS was performed in eleven patients at a mean of 9.4 years post-onset (Supplementary Table 1). Most had initiated EN (82%) and bladder catheterization (82%) before surgery. During follow-up, four patients (36%) died (cancer, sudden death, urinary tract infection, or heart failure); no deaths were attributed to aspiration pneumonia. Mean survival was 14.5 years, with some survivors exceeding 20 years under TIV. These long-term survivors maintained limited oral intake alongside EN according to individual preference.

## 4 Discussion

Recent multicenter data from the PROSPECT-M-UK cohort showed that gastrostomy was recommended in 13% and performed in 6.2% of patients with atypical parkinsonian disorders, with a median post-gastrostomy survival of 24 months [4]. Although not directly comparable because our EN definition included non-gastrostomy feeding, EN was used in 55% of our cohort, with a median post-EN survival of 4.3 years. These differences may partly reflect differences in patient selection, intervention timing, and care settings, including the long-term inpatient care provided at our institution.

EN alone does not completely prevent aspiration pneumonia caused by salivary stasis or reflux [1], [4]. This limitation highlights the potential complementary role of aspiration-prevention procedures such as LTS, which separate the airway from the digestive tract [7], [8]. In our small LTS subgroup, no deaths were attributed to pneumonia, and partial oral intake was maintained in some patients, although these findings should be interpreted cautiously. Regarding timing, although our study was not designed to determine an optimal time point, EN may be considered when objective swallowing assessment indicates that oral intake is becoming unsafe or insufficient, before recurrent aspiration pneumonia or severe malnutrition develops. Furthermore, recent literature emphasizes that decisions regarding EN and airway interventions should be integrated into structured advance care planning (ACP) early in the disease course, rather than initiated during acute emergencies [5], [12].

As shown in our Simon–Makuch curves, EN was generally introduced late in the disease course. In multivariable time-dependent Cox models, EN remained associated with lower mortality after adjustment for T/TIV, suggesting an additive contribution. The Fine–Gray analysis revealed a reversal of CIFs for death and T/TIV between groups (Fig. 1C). Patients receiving EN were more likely to undergo respiratory interventions before death, whereas those without EN often died before such interventions could be implemented. This suggests that the apparent benefit of EN reflects a multidisciplinary approach facilitating timely respiratory management, rather than nutrition alone.

Clinically, EN initiation aims to ensure nutrition and reduce eating-related distress [1], [4]. In our matched cohort, however, EN was associated with a significant reduction in sudden death, while pneumonia-related deaths showed only a non-significant downward trend (Table 1). This reduction may partly reflect the effects of tracheostomy and TIV on sudden death, as these interventions were more frequently implemented in the EN group. This pattern further supports the interpretation that the observed survival association with EN should be considered in the context of subsequent respiratory management rather than aspiration prevention alone.

The mean survival in our EN group (11.9 years) exceeded the 7–9 years reported elsewhere [3], [11]. This may partly reflect our specialized long-term inpatient setting and multidisciplinary management. However, center-specific practices may limit generalizability.

In our LTS subgroup, the mean survival was 14.5 years. The absence of pneumonia-related deaths aligns with previous reports suggesting that LTS may reduce aspiration and aspiration pneumonia [7], [8]. Clinically, LTS may complement EN and T/TIV by allowing safer supplemental oral intake. However, our subgroup was small and highly selected, with a predominance of younger patients with MSA-C, precluding causal inference.

LTS necessitates significant trade-offs, including the irreversible loss of phonation. Procedure choice depends on patient-specific factors, including aspiration severity, patient preferences, and care goals. Early, structured ACP is therefore important to balance aspiration control and the potential for oral intake against the loss of voice and other burdens associated with the procedure.

Beyond survival, the goal of EN and LTS is to support dignified, patient-centered living. Optimal management must balance survival with QoL, comfort, and autonomy. Future studies should prioritize longitudinal assessments of patient-reported QoL, caregiver burden, and communication ability to support ethically informed, individualized decision-making in advanced MSA.

## 5 Conclusion

EN was associated with longer survival in MSA, potentially reflecting its integration within a comprehensive nutritional and respiratory care pathway. In a small LTS subgroup, no pneumonia-related deaths occurred and limited oral intake was maintained in some patients. These findings support individualized nutritional and respiratory strategies within structured ACP.

## 6 Limitations

### 6.1 Single-Center design and generalizability

This study was conducted at a single specialized hospital providing long-term inpatient care, which may limit generalizability to outpatient or community settings. Patient selection and intervention timing likely reflect institutional protocols. Although calendar-time effects over the 25-year study period cannot be excluded, sensitivity analyses showed no significant interaction between EN and onset era.

### 6.2 Retrospective design and residual confounding

Although we mitigated immortal time and selection biases using time-varying covariates and propensity-score methods, unmeasured confounding remains possible. Furthermore, objective nutritional indices, including the Controlling Nutritional Status score, Geriatric Nutritional Risk Index, and body mass index, could not be systematically evaluated at EN initiation because of the retrospective design, limiting assessment of the relationship between nutritional status and survival. Additionally, individual socioeconomic factors, such as household income and informal caregiving capacity, were unavailable and could not be adjusted for, although public medical expense support and long-term inpatient care may have reduced socioeconomic barriers to EN.

### 6.3 Lack of QoL and ACP evaluation

This study focused on survival outcomes and did not assess QoL, symptom relief, or patient satisfaction. Documentation of ACP discussions was also limited. Future studies should incorporate structured longitudinal assessments of QoL and ACP to better evaluate patient-centered outcomes in advanced MSA.

## CRediT authorship contribution statement

**Katsuya Nishida:** Writing – original draft, Methodology, Investigation, Formal analysis, Data curation, Conceptualization. **Kento Sakashita:** Writing – review & editing. **Naonobu Futamura:** Writing – review & editing.

## Declaration of competing interest

The authors declare that they have no known competing financial interests or personal relationships that could have appeared to influence the work reported in this paper.

## Footnotes

Appendix A Supplementary data to this article can be found online at https://doi.org/10.1016/j.prdoa.2026.100499.

## References

[1] Calandra-Buonaura G., Alfonsi E., Vignatelli L. (2021). Dysphagia in multiple system atrophy: consensus statement on diagnosis, prognosis and treatment. Parkinsonism Relat. Disord..

[2] Do H.J., Seo H.G., Lee H.H. (2020). Progression of oropharyngeal dysphagia in patients with multiple system atrophy. Dysphagia.

[3] Wada T., Shimizu T., Asano Y. (2024). Early-onset dysphagia predicts short survival in multiple system atrophy. J. Neurol..

[4] Kobylecki C., Chelban V., Goh Y.Y. (2024). Frequency and outcomes of gastrostomy insertion in a longitudinal cohort study of atypical parkinsonism. Eur. J. Neurol..

[5] O’Shea N., Lyons S., Higgins S. (2023). Neurological update: the palliative care landscape for atypical parkinsonian syndromes. J. Neurol..

[6] Nishida K., Sakashita K., Yamasaki H. (2022). Impact of tracheostomy invasive ventilation on survival in Japanese patients with multiple system atrophy. Parkinsonism Relat. Disord..

[7] Taguchi E., Kobayashi Y., Tsuzuki H. (2022). Effect of aspiration prevention surgery in three patients with multiple system atrophy who have been hospitalized for aspiration pneumonia. Rinsho Shinkeigaku.

[8] Ueha R., Nito T., Sakamoto T. (2016). Post-operative swallowing in multiple system atrophy. Eur. J. Neurol..

[9] Gilman S., Wenning G.K., Low P.A. (2008). Second consensus statement on the diagnosis of multiple system atrophy. Neurology.

[10] Kanda Y. (2013). Investigation of the freely available easy-to-use software “EZR” for medical statistics. Bone Marrow Transplant..

[11] Nishida K., Sakashita K., Futamura N. (2025). Decision-making trends in therapeutic interventions for multiple system atrophy: a 24-year retrospective study. Mov. Disord. Clin. Pract..

[12] Breitegger C., Krismer F., Lorenzl S., Schrag A., Jahn B., Knoflach-Gabis A., Gabl C., Prajczer S., Fanciulli A., Schmidhuber M. (2024). Advance care planning in multiple system atrophy: ethical challenges and considerations. Clin. Auton. Res..

