# Supplementary material for: Survival benefit of enteral nutrition and airway interventions in multiple system atrophy
Source: Clin Park Relat Disord. 2026 Sep 9;15:100499. doi: 10.1016/j.prdoa.2026.100499 (PMC13587071; doi:10.1016/j.prdoa.2026.100499)
Supplement: Supplementary file 1 [file mmc1.docx]

**Supplementary Table 1**.

Clinical characteristics and outcomes of the laryngotracheal separation (LTS) subgroup (*n* = 11).

|  | **LTS subgroup**  **(*n* = 11)** |
| --- | --- |
| **Patient characteristics** |  |
| Age at onset, y | 51.8 (8.4) |
| Sex, male, *n* (%) | 4 (36) |
| Clinical phenotype, MSA-C, *n* (%) | 10 (91) |
| **Interventions, *n* (%)** |  |
| Urinary catheterization | 9 (82) |
| Enteral nutrition | 9 (82) |
| Tracheostomy | 11 (100) |
| Tracheostomy invasive ventilation | 4 (36) |
| **Clinical outcomes** |  |
| Time from onset to LTS, y | 9.4 (3.5) |
| Duration of hospitalization, y | 5.4 (5.1) |
| Overall survival time, y | 14.5 (6.1) |
| Death, *n* (%) | 4 (36) |
| **Causes of death, *n* (%)** |  |
| Aspiration pneumonia | 0 (0) |
| Colon cancer | 1 (9) |
| Heart failure | 1 (9) |
| Sudden death | 1 (9) |
| Urinary tract infection | 1 (9) |

**Footnote:** Data are presented as mean (SD) or *n* (%) unless otherwise indicated.

**Abbreviations**: LTS, laryngotracheal separation.
